# Brain oscillatory activity during motor preparation: effect of directional uncertainty on beta, but not alpha, frequency band

**DOI:** 10.3389/fnins.2015.00246

**Published:** 2015-07-21

**Authors:** Charidimos Tzagarakis, Sarah West, Giuseppe Pellizzer

**Affiliations:** ^1^Brain Sciences Center, Veterans Affairs Health Care ServiceMinneapolis, MN, USA; ^2^Department of Neuroscience, University of MinnesotaMinneapolis, MN, USA; ^3^College of Biological Sciences, University of MinnesotaMinneapolis, MN, USA

**Keywords:** magnetoencephalography, directional uncertainty, motor preparation, sensorimotor cortex, alpha-band, beta-band

## Abstract

In time-constraint activities, such as sports, it is advantageous to be prepared to act even before knowing precisely what action will be needed. Here, we studied the relation between neural oscillations during motor preparation and amount of uncertainty about the direction of the upcoming target. Ten right-handed volunteers participated in a cued center-out task. A brief visual cue identified the region of space in which the target would appear. Three cue sizes were used to vary the amount of information about the direction of the upcoming target. The target appeared at a random location within the region indicated by the cue, and the participants moved a joystick-controlled cursor toward it. Time-frequency analyses showed phasic increases of power in low (delta/theta: <7 Hz) and high (gamma: >30 Hz) frequency-bands in relation to the onset of visual stimuli and of the motor response. More importantly in regard to motor preparation, there was a tonic reduction of power in the alpha (8–12 Hz) and beta (14–30 Hz) bands during the period between cue presentation and target onset. During motor preparation, the main source of change of power of the alpha band was localized over the contralateral sensorimotor region and both parietal cortices, whereas for the beta-band the main source was the contralateral sensorimotor region. During cue presentation, the reduction of power of the alpha-band in the occipital lobe showed a brief differentiation of condition: the wider the visual cue, the more the power of the alpha-band decreased. However, during motor preparation, only the power of the beta-band was dependent on directional uncertainty: the less the directional uncertainty, the more the power of the beta-band decreased. In conclusion, the results indicate that the power in the alpha-band is associated briefly with cue size, but is otherwise an undifferentiated indication of neural activation, whereas the power of the beta-band reflects the level of motor preparation.

## Introduction

While doing activities that require prompt and accurate motor responses –for example, when playing sports or video games– it is advantageous to be prepared to react even before knowing precisely what the required action is going to be. For example, skilled tennis players prepare to respond before their opponents hit the ball by anticipating the most likely shot (Shim et al., 2005). This early motor preparation leads to a shorter latency of response when the action needs to be executed (Rosenbaum, 1980; Ghez et al., 1997) which is beneficial in fast action activities. However, the level of motor preparation that can be achieved in advance depends on several factors, among which is the contextual information that indicates what responses are likely to be needed. For example, in reaching tasks the latency of response decreases progressively with the degree of uncertainty about the direction of the upcoming target (Bock and Arnold, 1992; Bock and Eversheim, 2000; Pellizzer and Hedges, 2003, 2004). These results suggest that the amount of motor preparation changes gradually as a function of target uncertainty. However, how this function of motor preparation is translated into brain mechanisms is not well-understood.

It has been known for many years that brain oscillatory activity changes, not only during motor execution, but during motor preparation as well (Jasper and Penfield, 1949). Specifically, motor-related processing is associated with a decrease in power of alpha (8–12 Hz) and beta (14–30 Hz) oscillations (Pfurtscheller and Lopes da Silva, 1999; Neuper et al., 2006). The reduction of power is often referred to in the literature as desynchronization (Pfurtscheller and Lopes da Silva, 1999). This decrease in oscillatory activity in either the alpha- or the beta-band is considered to be a marker of motor processing (Pfurtscheller et al., 1994; Babiloni et al., 1999; McFarland et al., 2000; Yang et al., 2015) and are frequently used as intent-to-move control signals in brain-computer interfaces (McFarland et al., 2006; Neuper et al., 2009; Bai et al., 2011; Yi et al., 2013). However, there is also evidence that changes in alpha and beta oscillations reflect different brain mechanisms associated with motor processing (Cheyne, 2013; Tan et al., 2013).

How neural oscillations, particularly those in the alpha- and beta-bands, reflect the degree of motor preparation is unclear. In a previous study we had shown that the decrease of beta-band oscillations during movement preparation was modulated by the amount of uncertainty about the direction of target to be reached (Tzagarakis et al., 2010). The greater the directional uncertainty was, the less the power in the beta-band decreased during motor preparation. In that study, directional uncertainty was implemented using discrete cues that indicated the set of possible target locations. However, the visual cues remained present during the delay period of the task which confounded motor preparation and number of visual stimuli. In addition, the role of the alpha-band was not explored. Furthermore, we wanted to test the generality of the previous finding by implementing the degree of directional uncertainty using cues that identified the range of possible target direction rather than sets of discrete locations. For these reasons, we investigated how the alpha- and beta-band brain oscillatory activity during motor preparation in a reaching task is affected by the angular range of possible target directions. We found that, even though both alpha and beta oscillations showed a sustained decrease in power during motor preparation, only the beta-band reflected the level of motor preparation.

## Materials and methods

### Participants

Ten right-handed volunteers participated in the study (5 males and 5 females; mean age = 29 years; age range = 22–41 years). All participants had normal or corrected-to-normal vision. They had no reported history of neurological or psychiatric disorder, no reported substance abuse, and no reported use of tobacco for at least 1 month prior to the recording session. All participants provided informed consent prior to participating in the study and received monetary compensation for their participation. The experimental protocol was approved by the Institutional Review Board of the Minneapolis VAHCS.

### Task

**Figure 1:**
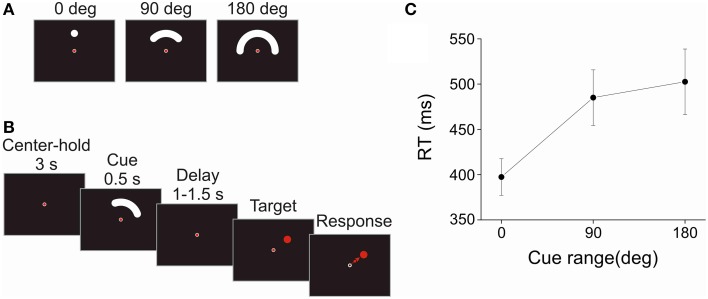
**Task and reaction time. (A)** Cues of different directional range (0°, 90°, or 180°) indicated the area in which the upcoming target could appear. These cues were presented in any random direction around the center. **(B)** Schematic sequence of events of the instructed-delay task. To start a trial, the subjects fixated and held the joystick-controlled cursor in the center of the display for 3 s. Then, a cue -instructing the subjects about the possible location of the upcoming target- was presented for 0.5 s. The figure shows a trial with the 90° cue range. After a randomly variable 1–1.5 s delay period, the target was presented at a random location within the range indicated by the cue. The subjects had to move the cursor quickly and accurately from the center toward the target. In addition, the subjects were instructed to fixate the center of the screen from the center-hold period to the onset of the target. An inter-trial interval (ITI) of 3 s followed each trial. **(C)** The reaction time (RT) was defined as the duration between the onset of the target and the onset of the response, which was determined by the exit of the cursor from the center window. The harmonic mean of RT is plotted against cue range. The error bars indicate the standard error of the mean across subjects (*N* = 10). RT increased significantly with the directional range of the cue.

The task was an instructed-delay reaching task in which a brief (0.5 s) visually presented cue indicated the range of directions in which the upcoming target could appear. Three cue sizes were used corresponding to target directional uncertainty ranges of 0°, 90°, and 180° (see Figure 1A). These cues were presented in any direction around the center of the screen. Trials with different cue range and direction were randomly shuffled. The target appeared at an equiprobable random direction within the range identified by the 90° or 180° cue. By definition with the 0° cue, the target could appear only at a single location within the cue. Each cue size was presented at least 60 times. When an error occurred (see below), the trial was reinserted randomly in the sequence of remaining trials, so that each subject had a complete set of 180 (i.e., 3 cue sizes × 60 repetitions) correct trials in the task. A block of 12 practice trials preceded the actual recording.

Subjects performed the task with their right hand. A trial was initiated by placing a joystick-controlled cursor within a small circular window (diameter = 1° of visual angle) in the center of the display for a 3 s center-hold period, and by fixating the center. Fixation had to be within 2° from the center to be valid. After the center-hold period, the cue was presented for a fixed period of 0.5 s, at 4° of visual angle from the center. The cue period was followed by a random delay period uniformly distributed between 1 and 1.5 s, after which the target appeared. The target was a red disc (diameter = 2° of visual angle) that appeared at a random location within the region previously subtended by the cue. The subjects were instructed to fixate the center of the display until the presentation of the target. Direction of gaze was monitored on-line using a video-based tracking system (ISCAN ETL-400, ISCAN Inc., Woburn, MA). If the subjects blinked or did not maintain fixation, the trial was aborted. When the target appeared, the subjects had to move the cursor quickly from the center to the target. The minimum time on target was set at 100 ms which constrained participants to slow down when approaching the target but did not need to stay on it. The trajectory of the cursor had to remain within virtual boundaries tangent to the center window and the target; otherwise the trial was counted as a movement direction error. The reaction time (RT) was defined as the time elapsed between the onset of the target and the exit of the cursor from the center window. RTs shorter than 100 ms or longer than 1500 ms were counted as RT errors. The movement time (MT) was defined as the time between when the cursor exited the center window and when it entered the target. MTs greater than 1500 ms were considered MT errors. When any of these errors occurred, the trial was presented again at a random position in the sequence of the remaining trials. An auditory signal at the end of the trial provided an additional feedback regarding whether the trial was correct or not. An inter-trial interval of 3 s separated each trial. A schematic representation of a trial in the task is shown in Figure 1B.

### MEG recordings

Data acquisition was performed similarly to a previously reported experiment (Tzagarakis et al., 2010). The subjects were lying supine on a bed inside of a magnetically shielded room with their head in the detector helmet. The visual stimuli and joystick-controlled cursor were projected on a screen about 60 cm in front of the subject using a LCD video projector (Sony VPL-PX20) located outside of the shielded room. The joystick (model M11C0A9F customized for MEG compatibility, CH Products, Vista CA, USA) was positioned on the bed next to the subject's right hip so that it could be manipulated comfortably with the hand.

Neuromagnetic signals were recorded using a 248-channel whole-head MEG system equipped with first-order axial gradiometers (Magnes 3600 WH, 4-D Neuroimaging, San Diego CA, USA). The signals were low-pass filtered (DC-400 Hz) and sampled at a rate of 1017.25 Hz. An electrooculogram (EOG) was recorded in addition to the video-based eye-tracking signal to help identify epochs contaminated by eye movements or eye blinks. In addition, the onset time of the visual stimuli (cue and target) on the screen was measured with a photodiode. The video-based eye-tracking, EOG, photodiode and joystick signals were recorded in auxiliary channels of the MEG system to ensure their synchronization with the MEG recordings. Five small coils were attached on the subject's head to measure the position of the head relative to the detector array at the beginning and end of the recording session. The head shape of each subject was digitized using a 3-D digitizer (Fastrak, Polhemus, Colchester VT, USA). In addition, the position of three fiducial points (nasion, left, and right pre-auricular points) was also digitized.

### MRI

We obtained head magnetic resonance images (MRI) from the participants for the co-registration of MEG data and brain anatomy. T1-weighted images were acquired with a 3-dimensional multiplanar gradient echo sequence using a 3 Tesla system (Achieva, Philips Medical Systems, Andover MA, USA; repetition time = 8.0744 ms; echo time = 3.695 ms; flip angle = 8°; field of view = 240 × 240 mm; matrix = 256 × 256 pixels; slice thickness = 1 mm). The volume of the scan extended from the top of the head to the bottom of the cerebellum and included all fiducial points. The co-registration of MRI and MEG data was performed using the head shape and the fiducial points. MRIs were obtained from nine out of ten participants. The missing set of MRIs was substituted by selecting a set from another participant with a similar head shape.

### Pre-processing of MEG data

**Figure 2:**
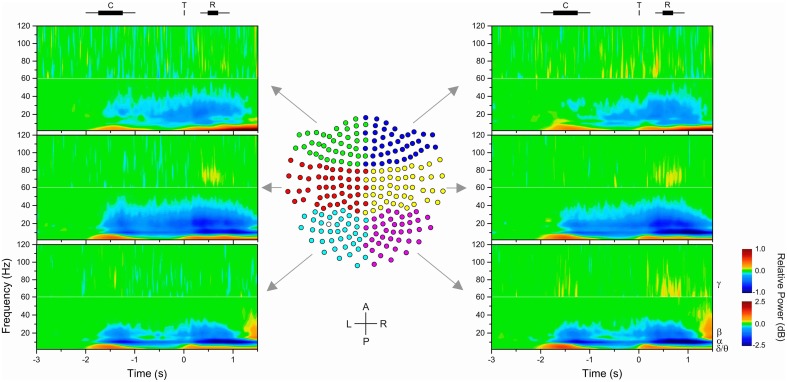
**Time-frequency maps of MEG gradiometers**. Data were grouped to simplify the presentation in six groups of sensors (left/right × posterior/middle/anterior), as shown in the center plot of the 2D projection of the 248-MEG sensor array. One gradiometer (empty circle) in the left-posterior (cyan) group was malfunctioning and discarded from the analyses. The time-frequency maps show the change in power from baseline of target-aligned data (*t* = 0 s). Power was estimated with a 10 ms time resolution and 2.5 Hz frequency resolution. However, color mapping was plotted following contour levels. Average (rectangle) and range (line) of task events are shown on top of the figure (C: cue, T: target; R: response). Different power scales were used for data below and above 60 Hz to improve the visibility of changes in power. Examination of the plots reveals differences in time-course and spatial distribution of different frequency bands.

MEG data were analyzed with custom-made MATLAB (Mathworks Inc., Natick MA) programs using the open-source Fieldtrip toolbox (Oostenveld et al., 2011). One left-posterior gradiometer was malfunctioning and discarded from all analyses (see Figure 2). Signals from reference sensors were used to subtract background noise from the neuromagnetic data using a 4-D-Neuroimaging algorithm implemented in Fieldtrip. In addition, trials contaminated by electronic artifacts (“SQUID jumps”), eye movements, eye blinks, or muscle activity were detected using a data-adaptive threshold and discarded. Cardiac artifacts were extracted using independent component analysis and removed. Finally, the data were detrended and an anti-aliasing low-pass filter was applied before resampling at 256 Hz to reduce the size of the files.

### MEG data analyses at the sensor level

Time-frequency power maps of cue-, target-, and movement-aligned data were computed using Morlet wavelets on a sliding window of 7-cycle width in 10 ms steps and 2.5 Hz resolution. Power in each frequency bin was computed relative to its baseline and expressed in dB. The baseline window was defined as −1000 to 0 ms for cue-aligned data, −3000 to −2000 for target aligned data, and −4000 to −3000 for movement aligned data.

### MEG data analyses at the source level

Analyses of MEG data at the source level were performed using adaptive beamformers. The localization of neural sources was estimated using the Dynamic Imaging of Coherent Sources (DICS) approach (Gross et al., 2001), whereas the time-varying power at sources identified with DICS was computed using the Linearly Constrained Minimum Variance (LCMV) approach (Van Veen et al., 1997). These beamformers estimate source power using spatial filters that minimize output variance with the constraint of unit gain at one source while minimizing signal gain from all other sources.

For each subject, MRIs were segmented in order to create a single-shell model of the brain surface (Nolte, 2003). The brain volume was divided in a regular grid of 8 mm voxels normalized into Montreal Neurological Institute (MNI) brain space. Lead fields were computed for dipoles at each grid location. The spatial filter coefficients were computed using regularized matrix inversion (5% regularization parameter). The contribution of the dipoles along the largest eigenvector was computed using singular value decomposition.

Localization of neural sources was computed by contrasting data from the delay period (1 s window preceding target onset) with data from the baseline period. The cross-spectral density matrix (for DICS) was computed between each pair of MEG sensor using pre-processed data from both periods jointly. The cross-spectral density matrix was computed using multitaper Fourier transform. For these calculations, the alpha-band was defined from 8 to 12 Hz, whereas beta-band was defined from 14 to 30 Hz. Power estimates were computed for each period separately and log-transformed before statistical analysis. Data from all subjects were analyzed by contrasting power estimates in the delay and baseline periods. Statistical significance was evaluated using a non-parametric cluster-based permutation test (Maris and Oostenveld, 2007), with a voxel-level threshold at *p* = 0.015 (two-tailed) and a corrected cluster-level significance at *p* = 0.05. The choice of the voxel-level threshold is to some degree an arbitrary one which, however, does not affect the validity of the cluster-level significance (Maris and Oostenveld, 2007). Here, we constrained the voxel-level threshold to be the same for both bands and aimed at preserving enough spatial extent for further analysis.

### Time-varying power at the source level

For each band of interest (alpha: 8–12 Hz; beta: 14–30 Hz) we estimated the time-varying power of each significant voxel. The data were first band-pass filtered (Butterworth filter) before LCMV beamforming. The signal time series for each significant voxel was obtained by multiplying the channel data with the LCMV spatial filter which was computed using the cross-variance matrix of pre-processed channel data (see Pre-processing of MEG data, above). Time-varying power for each voxel was then estimated as the squared modulus of the analytic signal computed using the Hilbert transform. The time-series were averaged across trials and normalized to baseline.

### Multidimensional analyses of the time-series

The time-series of relative power of all significant voxels for a specific frequency band have different degrees of similarity regarding their profile and amplitude. Differences in time-series reflect presumably variations in the involvement and/or the functional role of the brain source. For these reasons, we compared and classified the time-varying power from each voxel within the significantly activated area based on their similarity/dissimilarity. More specifically, the time-series were analyzed using Ward's hierarchical clustering method based on the Euclidean distance (Johnson and Wichern, 1998), implemented using MATLAB statistics toolbox. The time-series used in the clustering analysis were composed of concatenated time-series from each cue size condition (0°, 90°, and 180°) and from two time-periods: 0 to 1.5 s of the cue-onset-centered period, and −1 to 0 s of the target-centered period. The number of clusters retained for subsequent analyses was based on the Caliński-Harabasz criterion (Caliński and Harabasz, 1974), which has been shown to be one of the most reliable indexes for estimating the number of clusters in a dataset (Milligan and Cooper, 1985). The time-series of all voxels within a cluster were averaged to represent the time-varying power within that cluster.

In addition, we sought to examine the similarity of the power time-series across bands, as well as within and across clusters using a metric multidimensional scaling (MDS) analysis (Johnson and Wichern, 1998). To this end, we entered the time-series from all voxels with significant alpha- and beta-band activity in the MDS analysis (implemented using MATLAB function mdscale). Consequently, if a voxel was significant in both the alpha- and the beta-band, it was represented twice in the matrix of data. The Euclidean distance was calculated between all time-series to form a distance matrix. This matrix was then subjected to metric MDS using SStress as goodness-of-fit criterion. We performed the analysis for 2 and 3 dimensions and determined the best representation on the basis of the SStress outcome.

### Analysis of cluster-specific time-series

The time-series of relative power for each cluster, cue condition, and subject were smoothed using a thin plate spline (implemented using the R package mgcv). These time series were aligned either on cue presentation, target onset, or movement onset. Further analyses were performed on data averaged over 0.5 s epochs of interest selected to be representative of (1) the initial effect of cue presentation, (2) the ongoing effect during the delay period, and (3) the effect at movement onset. In this regard, we analyzed relative power averaged over (1) the 0.5 s period centered on the time of maximum desynchronization following cue onset; (2) the 0.5 s period from −0.6 to −0.1 s preceding target onset; and (3) the 0.5 s period starting at movement onset.

We also examined whether there was any dependence between the level of beta-band desynchronization during the delay period or at movement onset and cue or target direction, respectively. For this analysis we divided the trials for each condition according to the direction of the center of the cue, or according to the direction of the target. In both cases the directions were binned into four quadrants, thus creating four different direction categories. We then tested the effect of direction in the brain region that had the strongest beta-band desynchronization (see cluster 1 in Results), separately for each of the 0°, 90°, and 180° conditions, during the delay and response periods of the task.

Finally, we analyzed the time at which the time-varying power reached a relatively stable level after cue presentation both across bands and across clusters within each band. To this end, we identified the first minimum or inflection point, whichever came first, after cue presentation using the 1st and 2nd time-derivatives of the time-series of relative power for each band, cluster, and subject. For one analysis, we also determined this time per cue size condition.

### Analysis of voxel association across bands and clusters

In order to test the independence of voxel membership across bands and clusters, we counted the number of voxels that belonged to each category (band × cluster) and created a 3 × 4 contingency table (Table 1). We subsequently tested the hypothesis of independence using Fisher's exact test (Agresti, 1992).

**Table 1:** **Contingency table of number of voxels in the alpha- and beta-band clusters**.

		**Alpha**				
		**No cluster**	**Cluster 1**	**Cluster 2**	**Cluster 3**	**Total**
Beta	No cluster	4418	226	157	196	4997
	Cluster 1	8	0	84	2	94
	Cluster 2	259	6	310	132	707
	Total	4685	232	551	330	5798

### Statistical analyses of behavioral measures and relative-power

Statistical analyses of the effects of experimental factors on behavioral measures and on relative power during periods of interest were performed using linear mixed models with compound symmetry covariance structure and subject as a random factor (implemented using the R package nlme with type III sum-of-squares). The analysis of number of errors was performed using Box-Cox transformed values to normalize the distribution and stabilize the variance (Osborne, 2010). The lambda exponent of the Box-Cox transformation was optimized to minimize the skewness of the distribution of number of errors.

## Results

### Behavioral results

#### Response time and movement time

There was a significant effect of cue size on RT [*F*_(2, 18)_ = 13.667, *p* < 0.001]. Figure 1C shows that RT increased sharply between the 0° and 90° cue size and only slightly between the 90° and 180° cue size. In contrast, there was no significant effect of cue size on MT [*F*_(2, 18)_ = 0.736, *p* = 0.493; Mean MT = 213 ms, Standard error of the mean (SEM) = 31 ms, *N* = 10]. The patterns of RT and MT results are consistent with those obtained in a previous psychophysical study in which a very similar task was used (Pellizzer and Hedges, 2004).

#### Errors

Twenty-one percent of all trials were errors. The largest category of errors was due to eye fixation errors or eye blinks during the cue and delay periods (45% of error trials). The other large category of errors was that of directional errors (33% of error trials). The analysis of the Box-Cox transformed number of errors did not show any significant effect of cue size for either eye fixation errors/eye blinks [*F*_(2, 18)_ = 1.624, *p* = 0.225] or directional errors [*F*_(2, 18)_ = 1.025, *p* = 0.379].

### MEG results

#### Sensor level

The examination of spectrograms at the sensor level showed that, as expected, the spectral characteristics of MEG signals changed during the task and that these changes varied progressively across the array of sensors. Spectrograms averaged across subjects and groups of sensors are illustrated in Figure 2. The spectrograms were averaged over six groups of contiguous sensors to simplify the presentation. Figure 2 shows that there was a phasic increase in power in relation to the onset of the cue, target, and response in the theta/delta (<7 Hz) as well as gamma (>30 Hz) bands, whereas there was a tonic decrease in power in the alpha (8–12 Hz) and beta (14–30 Hz) bands from the onset of the cue to the response period, which, most importantly, included the delay period. In addition, the topographical distribution of change in power differed between the alpha- and beta-bands: it was more prominent in the middle and posterior group of sensors for the alpha-band, and more prominent in the middle group of sensors for the beta-band. Consistent with previous studies, we found that only alpha and beta oscillations provided a sustained change from baseline during motor preparation, that is, after information about the direction of the upcoming target was provided and before the target appeared (Pfurtscheller and Lopes da Silva, 1999; Alegre et al., 2003; Cheyne, 2013; Tan et al., 2013).

#### Source of alpha- and beta-band power change during the delay period

**Figure 3:**
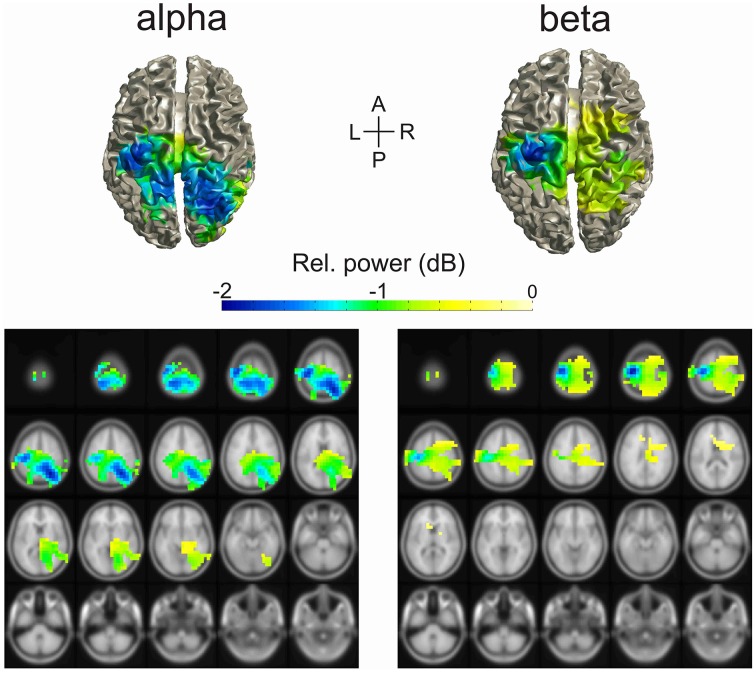
**Source localization of alpha and beta change in power during the delay period of the task**. The source of power change was estimated using the DICS beamformer method. The colored cortical regions were significantly different than baseline (cluster-based permutation test, *p* = 0.05).

We estimated the source of change of the alpha- and beta-bands during the delay period using DICS beamforming. The cortical areas significantly different from baseline are illustrated in Figure 3. The regions that contributed mostly to the decrease in power of the alpha-band were the left and right superior parietal cortices, including the precunei, the right inferior parietal cortex, and the left pre- and post-central gyri. Other regions of change in alpha-band included the right temporal lobe, the left inferior parietal cortex, and the left and right superior occipital gyri.

The regions that contributed the most to the decrease in power of the beta-band were the left pre- and post-central gyri. Other regions of change in beta-band included the right pre- and post-central gyri, the left, and right superior and inferior parietal cortices, the right mid frontal gyrus, the paracentral lobules, and the mid cingulate.

### Cluster analysis

**Figure 4:**
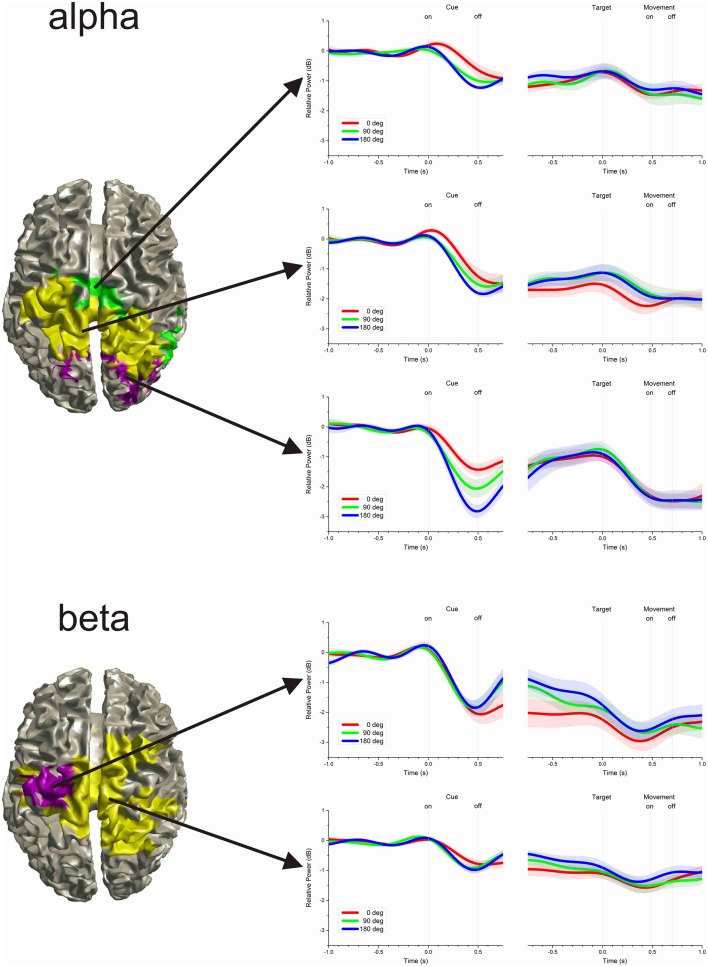
**Clusters of time-varying power**. The time-varying change in power in the alpha-band was differentiated into three clusters, whereas the beta-band was differentiated into two clusters. The left hand plots show the cortical projection of voxels attributed to each cluster. Even though no spatial information was included in the cluster analysis, voxels with similar time-varying profile were anatomically contiguous. The right hand plots show the average time-varying power for each cluster. The shaded region along the time-series indicates the inter-subject pointwise standard error of the mean.

The time-varying power of all significant voxels was compared using a cluster analysis to differentiate response profiles and localize their origin in the brain. The results showed that, even though no spatial information was used in the cluster analysis, voxels with similar time-varying profile were anatomically contiguous. The average time-varying change of power representative of each cluster and the cortical localization of the voxels attributed to each cluster are illustrated in Figure 4. For the alpha-band, the analysis differentiated three clusters: Cluster 1 was localized across the left and right occipital lobe; Cluster 2 was localized in the left and right inferior and superior parietal cortices, the left and right pre- and post-central gyri and precunei; Cluster 3 was localized in the right mid and superior temporal gyrus, the paracentral lobules, and mid cingulate. For the beta-band, the analysis differentiated two clusters: Cluster 1 was localized in the left pre- and post-central gyri; Cluster 2 was localized in the paracentral lobules, the precunei, the mid cingulates, the right post-central gyrus, the left, and right superior and inferior parietal cortices, and the right superior frontal gyrus.

### MDS analysis of power time-series

**Figure 5:**
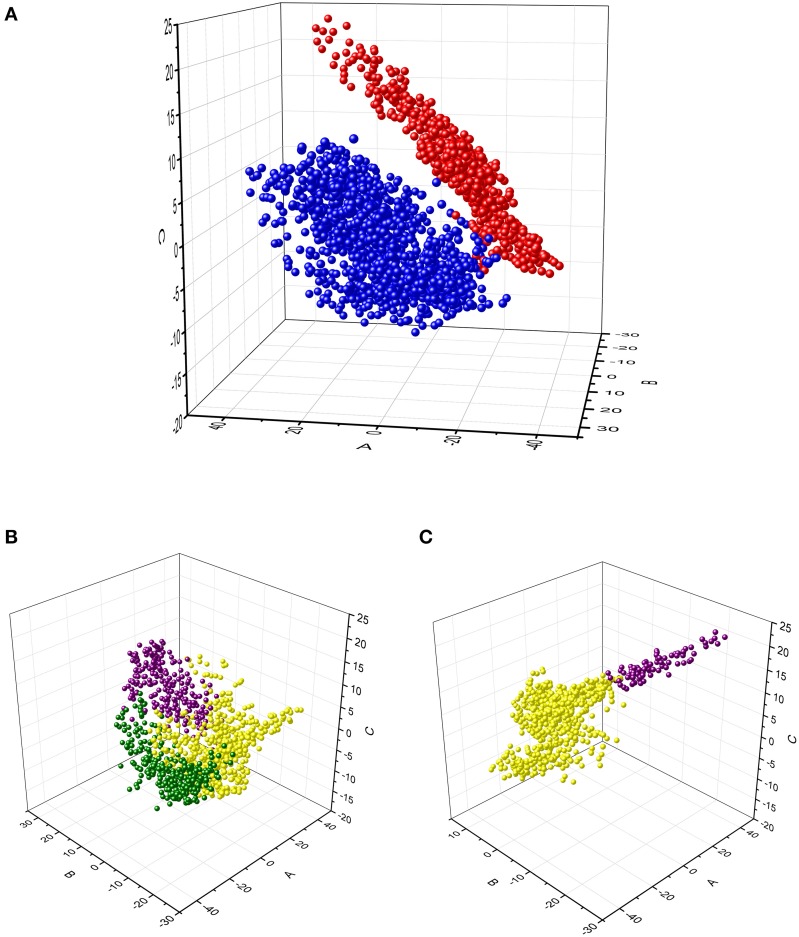
**Multidimensional scaling analysis of the time-series of alpha and beta relative power from each voxel within the significant brain regions**. **(A)** Projection of each data point in the 3-D MDS space. The data for alpha- (blue) and beta- (red) bands were colored differently to identify them in the MDS space. **(B,C)** Projection of the alpha-band and the beta-band data points, respectively. The data from the clusters obtained with the cluster analysis are identified by different colors (same as in Figure 4). These results illustrate that each cluster was located in a specific region within the MDS space, but data points varied continuously between those regions.

We analyzed the power time-series to examine whether the clusters identified above correspond to discrete groups of time-series profiles or whether they correspond to subdivisions within a continuum of variations of profiles. The MDS analysis was performed to achieve reduction of the multidimensional representation of time-series to 2 or 3 dimensions. The SStress value for two dimensions was 0.152, whereas it was 0.088 for three dimensions. SStress values < 0.1 indicate that the MDS dimensions provide a good representation of the data (Johnson and Wichern, 1998). For this reason, the 3-D MDS representation was selected for examination. The results are displayed in Figure 5. The MDS analysis showed that data from the alpha- and beta-band were well-separated (Figure 5A), whereas data from different clusters within band represented specific regions along a continuum of profiles of power time-series (Figures 5B,C).

### Analyses of alpha and beta power per epoch

**Figure 6:**
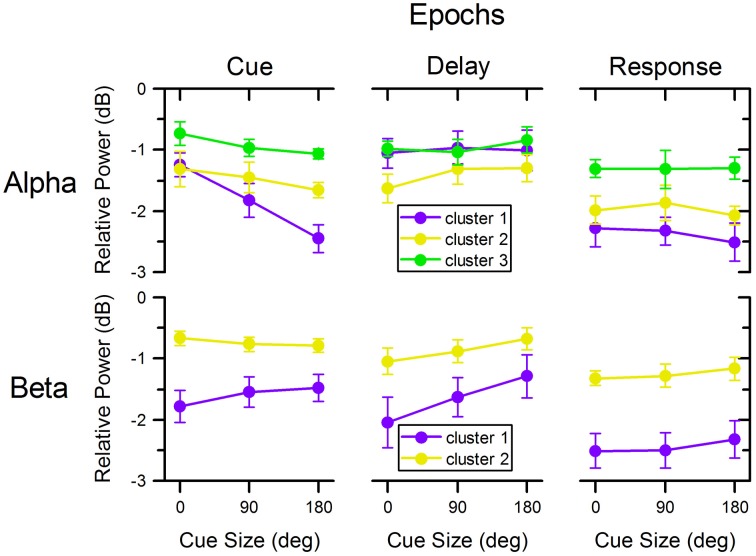
**Relative power across cue size, clusters, and task epochs**. Relative power of alpha and beta oscillations in the different clusters illustrated in Figure 4 across cue size and during different epochs of the task. Colors match those of corresponding clusters in Figures 4, 5.

Average relative power in alpha- and beta-bands during epochs of interest is plotted for each cue size and cluster in Figure 6. These data were analyzed using linear mixed models to determine the statistical significance of cluster, cue size, and their interaction.

### Alpha-band

Following cue presentation (0.5 s epoch centered on the time of minimum power following cue presentation: 0.490 s for cluster 1, 0.599 for cluster 2, and 0.603 s for cluster 3), there was a significant effect of cluster [*F*_(2, 72)_ = 32.321, *p* < 0.001], of cue size [*F*_(2, 72)_= 14.817, *p* < 0.001], as well as of their interaction [*F*_(4, 72)_ = 3.163, *p* = 0.019]. Separate linear mixed model analyses for each cluster indicated that there was a significant effect of cue size for cluster 1 [*F*_(2, 18)_ = 23.542, *p* < 0.001], but not for the other two clusters [cluster 2: *F*_(2, 18)_ = 1.603, *p* = 0.229; cluster 3: *F*_(2, 18)_ = 2.700, *p* = 0.094]. These analyses indicate that alpha power from cluster 1 changed significantly with cue size: the greater the cue size, the larger the deviation from baseline [linear polynomial contrast, *t*_(72)_ = −5.443, *p* < 0.001]. In addition, the decrease in power was significantly different across clusters, with cluster 1 having the largest overall deviation from baseline, followed by cluster 2 and then cluster 3 [*post-hoc* paired *t*-tests of cluster 1 vs. 2: *t*_(9)_ = −2.303, *p* = 0.047; cluster 1 vs. 3: *t*_(9)_ = −5.348, *p* < 0.001; cluster 2 vs. 3: *t*_(9)_ = −4.835, *p* = 0.001].

During the delay period preceding target onset, there was a significant effect of cluster [*F*_(2, 72)_ = 6.341, *p* = 0.003], but no effect of cue size [*F*_(2, 72)_ = 0.795, *p* = 0.455] or of cluster × cue size interaction [*F*_(4, 72)_ = 0.402, *p* = 0.806]. The significant effect of cluster resulted from the greater desynchronization of alpha in cluster 2, than in the other two clusters [*post-hoc* paired *t*-tests, cluster 1 vs. 2: *t*_(9)_ = 2.479, *p* = 0.035; cluster 1 vs. 3: *t*_(9)_ = −0.316, *p* = 0.760; cluster 2 vs. 3: *t*_(9)_ = −4.769, *p* = 0.001].

During the motor response, there was a significant effect of cluster [*F*_(2, 72)_ = 18.620, *p* < 0.001], but no significant effect of cue size [*F*_(2, 72)_ = 0.301, *p* = 0.741] or cluster × cue size interaction [*F*_(4, 72)_ = 0.138, *p* = 0.968]. The decrease of power was more pronounced for clusters 1 and 2 than for cluster 3 [*post-hoc* paired *t*-tests, cluster 1 vs. 2: *t*_(9)_ = −1.292, *p* = 0.228; cluster 1 vs. 3: *t*_(9)_ = −3.585, *p* = 0.006; cluster 2 vs. 3: *t*_(9)_ = −5.791, *p* < 0.001].

### Beta-band

Following cue presentation (0.5 s epoch centered on the average time of minimum power following cue presentation: 0.478 s for cluster 1 and 0.474 s for cluster 2), there was a significant effect of cluster [*F*_(1, 45)_ = 86.050, *p* < 0.001], but no significant effect of cue size [*F*_(2, 45)_ = 0.362, *p* = 0.698] or of cluster × cue size interaction [*F*_(2, 45)_ = 1.953, *p* = 0.154]. The effect of cluster was due to a greater reduction of beta power in cluster 1 than in cluster 2 [*post-hoc* paired *t*-test, *t*_(9)_ = −5.382, *p* < 0.001].

During the delay period, there was a significant effect of cluster [*F*_(1, 45)_ = 37.022, *p* < 0.001] and cue size [*F*_(2, 45)_ = 6.306, *p* = 0.004], but no significant effect of their interaction [*F*_(2, 45)_ = 0.801, *p* = 0.455]. The effect of cluster resulted from the greater desynchronization of beta power in cluster 1 than in cluster 2 [*post-hoc* paired *t*-test *t*_(9)_ = −4.123, *p* = 0.003], whereas the effect of cue size was due to the progressive change of beta power across cue size: the smaller the cue size, the greater the reduction of beta power during the delay period [linear polynomial contrast, *t*_(45)_ = 3.551, *p* < 0.001]. Separate linear mixed model analyses for each cluster showed that there was a significant effect of cue size for cluster 1 [*F*_(2, 18)_ = 5.986, *p* = 0.010] but only close to significance for cluster 2 [*F*_(2, 18)_ = 2.980, *p* = 0.076]. In addition, we found no significant effect of cue direction on cluster 1 beta desynchronization during the delay period for any of the three cue size conditions [0°: *F*_(3, 27)_ = 1.105, *p* = 0.364; 90°: *F*_(3, 27)_ = 0.696, *p* = 0.563; 180°: *F*_(3, 27)_ = 0.985, *p* = 0.415].

Finally, during the motor response the power of the beta-band was significantly affected by cluster [*F*_(1, 45)_ = 111.588, *p* < 0.001], but not by cue size [*F*_(2, 45)_ = 0.901, *p* = 0.414] or by cluster × cue size interaction [*F*_(2, 45)_ = 0.023, *p* = 0.977]. Like for the two preceding task epochs, the decrease in beta power during the motor response was more pronounced for cluster 1 than for cluster 2 [*post-hoc* paired *t*-test, *t*_(9)_ = −6.679, *p* < 0.001]. The direction of target had no significant effect on the power of the beta-band from cluster 1 in any of the cue size conditions [0°: *F*_(3, 27)_ = 1.581, *p* = 0.217; 90°: *F*_(3, 27)_ = 0.721, *p* = 0.548; 180°: *F*_(3, 27)_ = 2.754, *p* = 0.062].

### Timing of cue effect

The timing of the reduction of alpha- and beta-band power following cue presentation was analyzed using a linear mixed model with the factors band and cluster nested within band. The results showed that the timing was significantly different across bands [*F*_(1, 36)_ = 12.389, *p* = 0.001], as well as across cluster within band [*F*_(3, 36)_ = 5.130, *p* = 0.005]. To analyze the effect of cluster within band, we performed separate linear model analyses for each band with cluster as a factor. We found a significant difference in timing between alpha-band clusters [*F*_(2, 18)_ = 7.966, *p* = 0.003], but not between beta-band clusters [*F*_(1, 9)_ = 0.762, *p* = 0.405]. The effect of timing for alpha-band clusters was due to the earlier occurrence of power reduction in cluster 1 than in the other two clusters [*post-hoc* paired *t*-test, cluster 1 vs. 2: *t*_(9)_ = −4.653, *p* = 0.001; cluster 1 vs. 3: *t*_(9)_ = −3.285, *p* = 0.009; cluster 2 vs. 3: *t*_(9)_ = −1.080, *p* = 0.308].

In addition, as the alpha-band power in cluster 1 was significantly different across cue size conditions, we compared the timing of power reduction across cue size condition in that cluster and found no significant difference [*F*_(2, 18)_ = 0.010, *p* = 0.990].

Furthermore, we compared the timing of beta reduction in power to the timing in the alpha-band clusters. To this end, we collapsed the clusters that had no significant difference between them in the previous analysis. We found that there was no significant difference in timing between the beta-band clusters and the alpha-band cluster 1 [*post-hoc* paired *t*-test, *t*_(9)_ = −0.736, *p* = 0.480]. In contrast, the timing for the beta-band clusters preceded significantly those of cluster 2 and 3 of the alpha-band [*post-hoc* paired *t*-test, *t*_(9)_ = −5.039, *p* = 0.001].

### Voxel dependence across bands and clusters

Table 1 indicates the number of voxels in each band × cluster category. Fisher's exact test for independence was significant for the voxel contingency table (*p* < 0.001, 2-sided), thus rejecting the hypothesis of independence of the distribution of voxels with significant alpha- and beta-band activity.

## Discussion

We investigated the effect of angular uncertainty about the direction of the upcoming target on brain oscillatory activity during motor preparation. Target directional uncertainty was controlled by presenting a brief visual cue that identified the range of directions in which the target would appear. Cues of three different sizes were used corresponding to directional uncertainties of 0°, 90°, or 180°. The visual cue was presented for only a brief period and was followed by a delay period before the onset of the target. Crucially, during the delay period of the task there was no visual information differentiating the conditions, therefore any difference in neuronal oscillations between conditions can only be related to the information provided previously by the cue and maintained during motor preparation. The information provided by the cues did affect motor preparation as indicated by the effect on reaction time. We found that only the alpha- and beta-band oscillations had a tonic reduction in power during the delay period of the task (i.e., during motor preparation), but only the power of the beta-band was different across uncertainty conditions during that period.

### Sources of tonic change of alpha- and beta-band oscillations during motor preparation

The topography of the reduction of alpha-band oscillations during motor preparation differed from that of the beta-band, although both overlapped over the sensorimotor region contralateral to the responding hand (Figure 3). As expected from the overlap of the brain regions with alpha and beta-band activity, we found that the distribution of voxels across bands and clusters were not statistically independent. Similarly to what has been described in other studies, the reduction in alpha oscillations was localized over a relatively broad centro-posterior region that included the contralateral sensorimotor region as well as bilateral parietal and occipital areas, whereas the reduction in beta activity was more restricted and involved mainly the contralateral sensorimotor region (Pfurtscheller, 1981; Salmelin and Hari, 1994; Crone et al., 1998; Babiloni et al., 1999). This difference in topography implies that the alpha- and beta-band were associated with different functional processes during motor preparation. In addition, the fact that the region of the brain with significant change of power relative to baseline extended across several anatomical and functional brain areas suggested that time-series of power likely differed even within a frequency-band. In order to investigate the existence of potential differentiation of power time-series profiles within bands we performed multivariate analyses of the time-series from each voxel.

The cluster analysis of time-varying power provided a data-driven approach to classify the source of oscillatory change into clusters of time-series that differed in profile and/or amplitude. Although there was no spatial information entered in the cluster analysis, voxels with similar time-varying profiles were found to be anatomically segregated and not intermixed across the brain. Consequently, the cluster analysis provided a method to describe the functional neuroanatomy associated with motor preparation. The results of the analysis suggested that the brain region with significant change in beta-band power could be divided into two clusters, whereas the region with significant alpha-band change could be divided into three clusters (Figure 4). Although the results of the cluster analysis may vary depending on the method selected to group the data, they indicate nevertheless that there were different functional contributions across the brain regions involved in motor preparation. However, the separation of clusters from this analysis does not mean that the profiles of time-varying power in each cluster are necessarily discrete groups of time-series. In this respect, the MDS analysis showed that the time-series of the alpha- and beta-band were well-separated in 3-D multidimensional space, whereas time-series of different clusters within a specific band changed progressively from one region to another in the MDS space (Figure 5). Consequently, the MDS analysis indicated that even though the power of both the alpha- and beta-band decreased during motor preparation, their time-series profiles were distinct. In contrast, the time-series profiles within band varied continuously from the profile characteristics of one cluster to the profile characteristics of another cluster. The continuity of the variations of power time-series may indicate an underlying physiological continuity across brain regions; however, it may also indicate a limitation in the resolution of the source analysis.

### Time-varying power of the alpha-band

The analysis of time-varying power of alpha oscillations across the centro-posterior region that was significantly different from baseline during motor preparation showed clear differences in profiles (Figure 4, top). Specifically, the time-varying activity of the more posterior cortical area, over the parieto-occipital border, had a phasic reduction of power following the onset of the visual cue. This reduction of power preceded the reduction of power in the other alpha-band clusters and was similar in timing with the reduction of beta-band power. Furthermore, the phasic reduction of power in the more posterior area was differentiated by cue size: the wider the cue, the greater the reduction of alpha oscillations. However, this differentiation did not carry over during the delay period of the task, even though a tonic reduction of power remained present in that region during the delay period. An additional reduction of alpha oscillation occurred in this posterior area following the onset of the target. This pattern of results suggests that the reduction of alpha oscillations in the more posterior area was at least in part the effect of visual stimulation. However, even during the delay period of the task, when there was no visual cue, there was a tonic reduction of alpha oscillations which suggests that this brain region remained activated. This continuous activation of the parieto-occipital region during the delay period might reflect the expectation of the upcoming visual stimulus (i.e., the target). The reduction of alpha oscillations has been associated with visual and spatial attention processes (Klimesch, 2012; Jensen et al., 2014).

Importantly in regards to motor preparation, the cortical region that had the greatest reduction of alpha oscillations during the delay period of the task was the middle region that encompassed the parietal lobes bilaterally and the contralateral sensorimotor region. These alpha oscillations over the motor region have been referred to as mu rhythms (Gastaut, 1952; Pineda, 2005; Neuper et al., 2006). Not unexpectedly, this result implies that this brain region was the most active during motor preparation. However, the reduction of alpha oscillations was undifferentiated across cue size, which is consistent with the idea that it reflects a gating mechanism of task-relevant brain regions (Jensen and Mazaheri, 2010).

### Time-varying power of the beta-band

The analysis of the time-varying power of beta oscillations showed that there was a strong reduction of power over the sensorimotor region contralateral to the responding hand (Figure 4, bottom). This reduction of power spilled over to the other cerebral hemisphere. We checked whether the level of power of the beta-band was affected by the direction of the center of the cue or the direction of the target and found no significant relation during either the delay or the response period. This lack of directional effect on the power of the beta-band is consistent with the results of other studies (Waldert et al., 2008; Ince et al., 2010). However, the main effect of interest was that the reduction of beta oscillations during the delay period of the task scaled with the uncertainty about the direction of the upcoming target: the smaller the cue, the greater the reduction in beta oscillations. This is consistent with the results of a previous study in which the reduction of beta power scaled with the number of possible target directions (Tzagarakis et al., 2010). However, in that study the visual cues remained present during the delay period of the task which was a confounding factor. In contrast, in the current study the visual cue was presented briefly and no visual information differentiated the conditions during the delay period. Consequently, the difference in beta power during the delay period in the current study is unambiguously related to maintaining the information provided previously by the cue about the range of direction in which the upcoming target would appear. These results indicate that directional uncertainty about the upcoming target determines the level of reduction of beta oscillations during motor preparation. The different levels of beta power with cue size can explain the effect of cue size on reaction time. This effect on reaction time is similar to what was found in other studies using a similar cuing task (Bock and Arnold, 1992; Pellizzer and Hedges, 2004). The absence of effect of uncertainty on the power of alpha oscillations and its presence on the power of beta oscillations in the sensorimotor region suggests that the reduction of alpha is associated with an activation gating mechanism (Jensen and Mazaheri, 2010), sensory anticipation (Buchholz et al., 2014), or an attention process (Alegre et al., 2003; Tan et al., 2013), whereas the reduction of beta is more directly related to motor preparation (Alegre et al., 2003; Baker, 2007; Tan et al., 2013).

### Network underpinnings of alpha and beta oscillations in the sensorimotor region

The reduction of alpha and beta oscillations in the sensorimotor region is associated with an increase in fMRI-BOLD response in the same region (Formaggio et al., 2008; Ritter et al., 2009; Yuan et al., 2010). Although there is a vast variety of relations between the amplitude of beta oscillations and neuronal spiking activity at the single-cell level in the motor cortex (Canolty et al., 2012), at the neuronal population level the reduction of beta oscillations is associated with an increase in neuronal spiking activity (Spinks et al., 2008; Canolty et al., 2012). For these reasons, the reduction in power of cortical alpha and beta oscillations is considered to be indicative of neural activation (Neuper et al., 2006; Formaggio et al., 2008; Ritter et al., 2009; Yuan et al., 2010). It has long been thought that neuronal oscillations result from the local network architecture, in particular the recurrent inhibition of pyramidal neurons (Andersen and Eccles, 1962; Jasper and Stefanis, 1965), although large scale cortico-cortical and thalamo-cortical interactions are also likely to play a role in the characteristics and in the topography of alpha and beta oscillations (Lumer et al., 1997; Izhikevich and Edelman, 2008). The importance of inhibitory interneurons in regards to the modulation of beta oscillations has been demonstrated by the administration of benzodiazepines, which act as GABA_A_ agonists, to healthy individuals. It was found that benzodiazepines have little effect on alpha oscillations in the sensorimotor region, whereas they increase beta oscillations (Hall et al., 2010, 2011; Gaetz et al., 2011; Muthukumaraswamy et al., 2013). A corresponding drug-related response of sensorimotor alpha oscillations has yet to be found. In other words, alpha oscillations and beta oscillations in the sensorimotor region are in all likelihood modulated by different inhibitory mechanisms. Furthermore, the stimulation of pyramidal tract neurons resets alpha and beta oscillations, which suggests that pyramidal tract neurons are part of the networks generating those oscillations (Jackson et al., 2002). However, only beta oscillations are coherent with electromyography activity (Baker et al., 1999; Jackson et al., 2002). Consequently, pyramidal neurons are most likely part of the networks generating beta oscillations, whereas they may be only indirectly associated with the networks generating alpha oscillations (Jackson et al., 2002). The relation of pyramidal tract neurons with beta oscillations may be the reason why beta oscillations are found to be more closely associated with motor preparation than alpha oscillations.

## Conclusions

The results of this study show a tonic reduction of alpha and beta-band oscillations associated with motor preparation. The main source of change of alpha oscillations covered a wider region than that of beta oscillations, but both sources overlapped over the sensorimotor region contralateral to the responding hand. Importantly, only the change of beta oscillations was dependent on directional uncertainty: the less the directional uncertainty, the greater the beta power reduction.

In conclusion, the results suggest that in the sensorimotor cortex the power of alpha is an undifferentiated indication of neural activation, possibly related to sensory expectation, attention, and gating of information, whereas the power of beta oscillations is more directly related to motor processing and reflects the level of motor preparation.

### Conflict of interest statement

The authors declare that the research was conducted in the absence of any commercial or financial relationships that could be construed as a potential conflict of interest.
